# Protease Activity Profiling of Snake Venoms Using High-Throughput Peptide Screening

**DOI:** 10.3390/toxins11030170

**Published:** 2019-03-19

**Authors:** Konstantinos Kalogeropoulos, Andreas Frederik Treschow, Ulrich auf dem Keller, Teresa Escalante, Alexandra Rucavado, José María Gutiérrez, Andreas Hougaard Laustsen, Christopher T. Workman

**Affiliations:** 1 Department of Biotechnology and Biomedicine, Technical University of Denmark, 2800 Lyngby, Denmark; konskalogero@gmail.com (K.K.); andreas.treschow@bio.dtu.dk (A.F.T.); uadk@dtu.dk (U.a.d.K.); ahola@bio.dtu.dk (A.H.L.); 2 Instituto Clodomiro Picado, Facultad de Microbiología, Universidad de Costa Rica, San José 11501-2060, Costa Rica; teresa.escalante@ucr.ac.cr (T.E.); alexandra.rucavado@ucr.ac.cr (A.R.); jose.gutierrez@ucr.ac.cr (J.M.G.)

**Keywords:** snake venom proteinases, high-throughput, peptide substrates, proteinase activity, screening, modeling, enzymatic profile

## Abstract

Snake venom metalloproteinases (SVMPs) and snake venom serine proteinases (SVSPs) are among the most abundant enzymes in many snake venoms, particularly among viperids. These proteinases are responsible for some of the clinical manifestations classically seen in viperid envenomings, including hemorrhage, necrosis, and coagulopathies. The objective of this study was to investigate the enzymatic activities of these proteins using a high-throughput peptide library to screen for the proteinase targets of the venoms of five viperid (*Echis carinatus*, *Bothrops asper*, *Daboia russelii*, *Bitis arietans*, *Bitis gabonica*) and one elapid (*Naja nigricollis*) species of high medical importance. The proteinase activities of these venoms were each tested against 360 peptide substrates, yielding 2160 activity profiles. A nonlinear regression model that accurately described the observed enzymatic activities was fitted to the experimental data, allowing for the comparison of cleavage rates across species. In this study, previously unknown protein targets of snake venom proteinases were identified, potentially implicating novel human and animal proteins that may be involved in the pathophysiology of viper envenomings. The functional relevance of these targets was further evaluated and discussed. These new findings may contribute to our understanding of the clinical manifestations and underlying biochemical mechanisms of snakebite envenoming by viperid species.

## 1. Introduction

Snake venoms are highly complex mixtures of proteins and peptides, comprising enzymes, non-enzymatic proteins and polypeptides from a wide range of families, and other bioactive components [1,2]. Proteinases are prevalent enzymes in the venoms of snakes in the Viperidae family [3,4,5,6], and are often the main drivers of toxicity for viperid venoms. These enzymes are known to affect a large number of physiological pathways and response cascades, assisting in immobilization and death. In addition, venom proteinases play a role in digesting prey, as well as deterring predators [7]. Proteinases have the ability to cleave other proteins by the hydrolysis of peptide bonds, whereby these other proteins may become activated or deactivated. In turn, this may modulate different physiological processes involved in homeostasis [8].

Among the various families of venom proteinases, those present in snake venoms predominantly belong to snake venom metalloproteinases (SVMPs) and snake venom serine proteinases (SVSPs). SVMPs are known to contribute to the pathophysiology of envenomings by causing inflammation, hemorrhage, blistering, skin damage, coagulopathy, and tissue necrosis in prey and patients [9]. In addition, they contribute to the hemodynamic alterations associated with viperid snakebite envenomings as a consequence of hypovolemia [10]. SVMPs may affect the hemostatic system through various mechanisms, such as activating prothrombin or coagulation factor X, exhibiting fibrinolytic and fibrinogenolytic activities, or inhibiting platelet aggregation [11]. SVSPs are capable of interacting similarly with the processes and components of the coagulation cascade. These enzymes degrade fibrinogen or promote coagulation and platelet aggregation by activating or inactivating several coagulation factors [12]. In addition, SVSPs cleave plasma proteins to generate kinins and other bioactive agents [13].

In some cases, snake venom proteinases are highly similar to the human proteinases involved in normal physiological processes. Many SVSPs are considered thrombin-like enzymes due to their structural similarities with thrombin, their targets, and their involvement in coagulation processes [14]. SVSPs are classified in a mixed endopeptidases group (the “PA” clan), S01 family, subfamily A of proteolytic enzymes by the MEROPS classification system (https://www.ebi.ac.uk/merops/) [15]. SVMPs, on the other hand, exhibit high similarity with ADAM (a disintegrin and metalloproteinase) group of human metalloproteinases [16], and are also classified in the same proteinase family, according to the MEROPS database (clan MA, family M12, subfamily B). Due to these similarities, elucidating the mechanisms of action and the targets of snake venom proteinases may prove important in other fields of biomedical research.

SVMPs are divided into three major classes, depending on the structural domains present in the given SVMP. The first and the simplest class of SVMPs (P-I) contain only a single metalloproteinase domain in the mature protein. The second class (P-II) contains SVMPs with a disintegrin domain and a metalloproteinase domain. The third and most complex class (P-III) consists of SVMPs with a metalloproteinase domain, a spacer, a disintegrin-like domain, and a cysteine-rich domain. Additionally, several subclasses within each class exist [17]. SVSPs have been categorized into different functional subtypes, including thrombin-like enzymes, kallikrein-like or blood pressure-reducing enzymes, plasminogen activators, and platelet aggregation inhibitors or inducers [18]. For both proteinase types, substrate specificity is regulated by conserved residues in the enzyme binding pockets, with different variations between subfamilies.

Snake venom proteinase activity has traditionally been investigated by the purification of a selected enzyme, followed by biochemical characterization. In these studies, the enzymatic activity of proteinases is examined in assays using general protein substrates, such as azocasein [19], by zymography [20], or with known targets of these enzymes, such as fibrinogenolytic, procoagulant, and platelet aggregation assays [12,14,21,22]. Recent studies have focused on the substrate specificity of proteinases of interest by the proteomic identification of cleavage sites. Using established purification and mass spectrometry methods, substrate specificity models for said proteinases can be constructed [23,24].

Proteinase activity can be measured in homogeneous assays, relying on a physical change following substrate catalysis. In detection assays, the change is often assessed by measuring a fluorescent or chromogenic signal from a synthetic peptide substrate upon cleavage by the proteinase. Proteinases tolerate some degree of substrate modification, making the addition of reporter groups in synthetic peptides feasible. Fluorescence resonance energy transfer (FRET) assays can be used in cases where the integrity of the P’ residues (residues following the cleavage site) of the substrate must be preserved [25]. To enable these assays to have a positive indication of proteinase activity, a variation of the assay where an acceptor–donor pair that allows maximum overlap between emission and absorption are used. The acceptor acts as a quencher when in close proximity, e.g., before cleavage, with the measured emission being close to zero. Upon cleavage, the moiety is separated, and the fluorophore emission can be measured using fluorescence readers [26].

**Figure 1:**
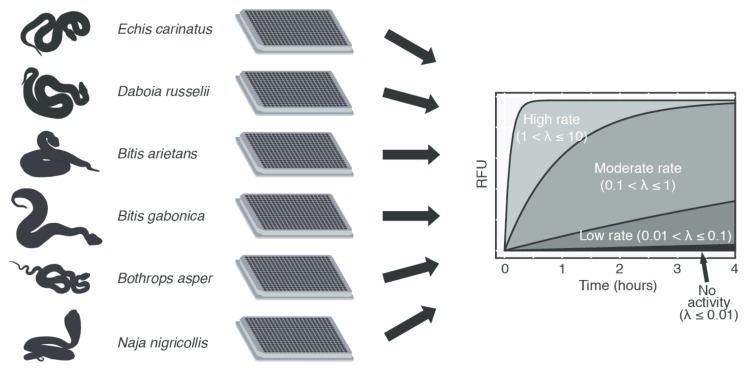
Overview of the experimental method used in this study. The snake venoms obtained were incubated with a peptide substrate set, yielding activity profiles for each peptide, across all snake venoms. The profiles were analyzed further to determine if the cleavage of a given target peptide was observed, and the rate of said cleavage (Section 2.3).

In this study, we demonstrate the use of the donor–acceptor detection method for the investigation of proteinase activity of selected crude venoms in a high-throughput setup. An overview of this method can be seen in Figure 1. With the use of a proteinase substrate set, consisting of substrates described to be cleaved by proteinases in the literature, we were able to gain insights into the proteinase activity of several medically relevant snake venoms. The total proteinase activity of these venoms was analyzed and compared. The use of this high-throughput substrate screening method also shows promise in unveiling novel target substrates of snake venom proteinases that could be involved in the pathophysiology of envenomings.

## 2. Results

### 2.1. Proteinase Activity Measurements Confirm Known Targets and Reveal New Substrate Sequences

Venoms from the snake species *Bitis gabonica*, *Bitis arietans*, *Daboia russelii*, *Echis carinatus*, *Bothrops asper* (family Viperidae), and *Naja nigricollis* (family Elapidae) were subjected to proteinase activity screening experiments. In total, 2160 activity profiles for these snake venoms against 360 different peptide targets were acquired. These profiles were examined for the magnitude of the proteinase activity, and the target specificity of the snake venoms. Based on the modeling parameter estimation of activity, and the number of cleaved substrates of these snake venoms in the substrate set as a whole, the highest activity was observed for the venoms of *E. carinatus*, *B. arietans,* and *B. asper*.

**Figure 2:**
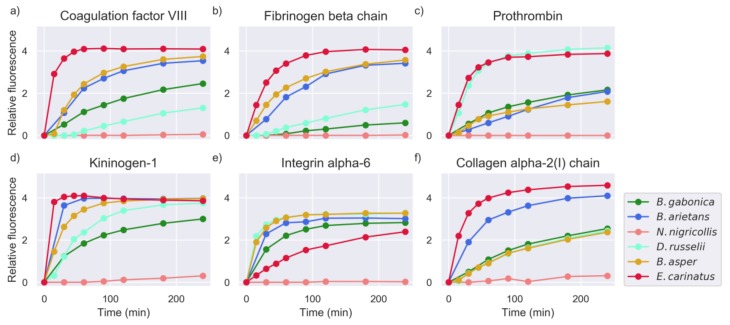
Measurements of selected substrates for all snake venoms (intensity measured in RFU: relative fluorescence units, see Section 4.3). The common name of the protein of origin is shown as a title of each plot. The Uniprot IDs and amino acid sequences of these substrates were: (**a**) FA8_HUMAN–PQLRMKNN, (**b**) FIBB_CHICK–IDARAHRP, (**c**) THRB_HUMAN–IDGRIVEG, (**d**) KNG1_HUMAN–SPFRSSRI, (**e**) ITA6_HUMAN–RPIPITAS, and (**f**) CO1A2_HUMAN–FYRADQPR.

The substrate screening set contained several peptide sequences originating from proteins known to be snake venom proteinase substrates. These included prothrombin, fibrinogen, other coagulation factors, integrins, and collagen-derived sequences. Examples of venom proteinase activity against these peptides are given in Figure 2.

**Figure 3:**
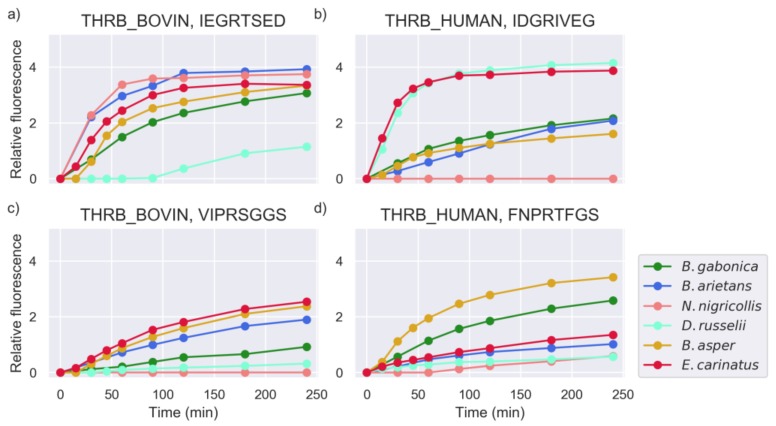
Measurements of substrates from human and bovine prothrombin (intensity measured in RFU). The plots are annotated with the Uniprot ID of the protein and the amino acid sequence of the substrate.

In a number of cases, different activity levels were detected for venoms against multiple peptides derived from the same proteins. This implies that the residue specificity plays a larger role in substrate recognition than the target selectivity. Figure 3 shows the different activities of the studied snake venoms against multiple candidate substrates originating from human and bovine prothrombin.

**Figure 4:**
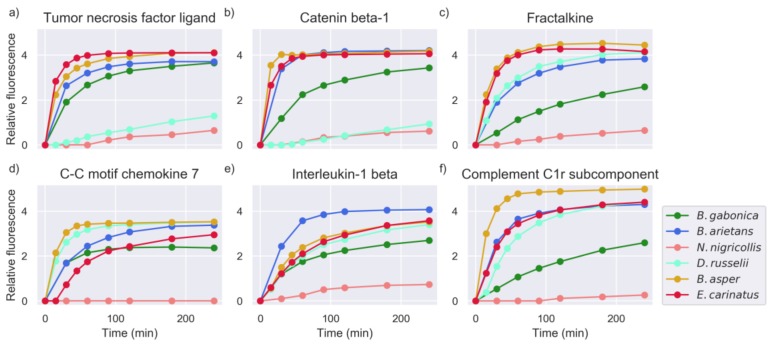
Measurements of selected substrates for all snake venoms (intensity measured in RFU). The common name of the protein of origin is shown as a title of each plot. The Uniprot IDs and amino acid sequences of these substrates were: (**a**) TNFL6_HUMAN–SLEKQIGH, (**b**) CTNB1_HUMAN–ADIDGQYA, (**c**) X3CL1_HUMAN–AATRRQAV, (**d**) CCL7_HUMAN–QPVGINTS, (**e**) IL1B_HUMAN–GPYELKAL, and (**f**) C1R_HUMAN–QRQRIIGG.

Apart from known snake venom proteinase substrates, high activity was also observed against other hitherto unknown substrates of venom proteinases. These peptides were derived from proteins that, to our knowledge, have no known cleavage sites for snake venom proteinases. These targets might be involved in snake venom pathophysiology, contributing to the toxic effects exerted by venom toxins or, alternatively, might reflect the overall digestive action of snake venom proteinases. Examples of these targets are shown in Figure 4.

The majority of snake venoms showed high activity for peptide substrates derived from proteins known to be inflammation mediators, such as tumor necrosis factor ligands, interleukins, kininogen, and complement system components. The exact influence of proteinase activity on these substrates remains unknown, but it is hypothesized that this activity may impact inflammatory processes around the bite site during envenoming.

### 2.2. Inhibition of Metalloproteinase Activity Provides Insights on Targets of Both Proteinase Families

In order to assess the proteinase activity of each of the two prominent proteinase families, SVMPs and SVSPs, additional experiments with the addition of the metalloproteinase inhibitor *o*-phenanthroline were carried out. The metal ion chelator *o*-phenanthroline has proven to be a potent inhibitor of all proteinases of the metalloproteinase family, due to its mechanism of action depriving the catalytic ions from these proteinases. The venoms that showed high activity in the original experiments (namely *E.carinatus*, *B. arietans* and *B. asper*) were incubated with *o*-phenanthroline prior to the peptide screenings (see Section 4.2).

**Figure 5:**
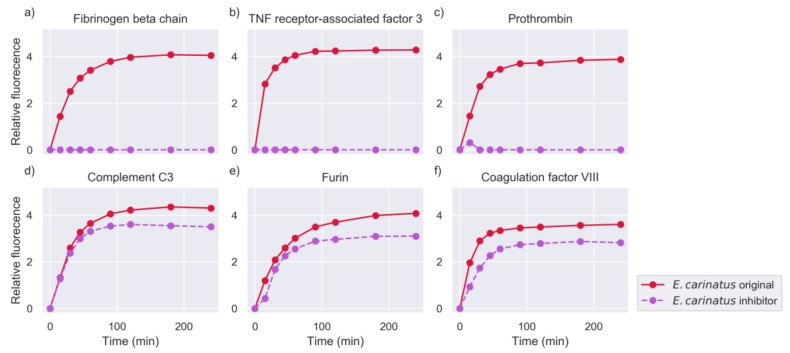
Measurements of selected substrates for the venom of *E. carinatus*, in the whole venom experiment (original) and in the metalloproteinase inhibition experiment (intensity measured in RFU). The common name of the protein of origin is shown as a title of each plot. The Uniprot IDs and amino acid sequences of these substrates were: (**a**) FIBB_CHICK–IDARAHRP, (**b**) TRAF3_HUMAN–EEADSMKS, (**c**) THRB_HUMAN–IDGRIVEG, (**d**) CO3_HUMAN–GLARSNLD, (**e**) FURIN_HUMAN–FWHRGVTK, and (**f**) FA8_HUMAN–IEPRSFSQ.

**Figure 6:**
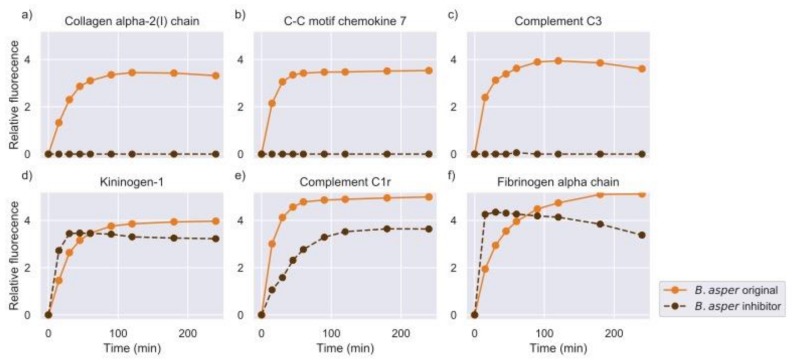
Measurements of selected substrates for the venom of *B. asper*, in the whole venom experiment (original) and in the metalloproteinase inhibition experiment (intensity measured in RFU). The common name of the protein of origin is shown as a title of each plot. The Uniprot IDs and amino acid sequences of these substrates were: (**a**) CO1A2_HUMAN–GPQGLLGA, (**b**) CCL7_HUMAN–QPVGINTS, (**c**) CO3_ONCMY–LLSRSEED, (**d**) KNG1_HUMAN–SPFRSSRI, (**e**) C1R_HUMAN–QRQRIIGG, and (**f**) FIBA_HUMAN–GGVRGPRV.

**Figure 7:**
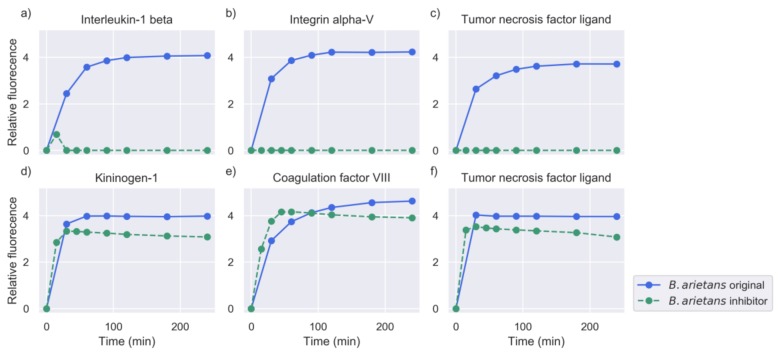
Measurements of selected substrates for the venom of *B. arietans*, in the whole venom experiment (original) and in the metalloproteinase inhibition experiment (intensity measured in RFU). The common name of the protein of origin is shown as a title of each plot. The Uniprot IDs and amino acid sequences of these substrates were: (**a**) IL1B_HUMAN–GPYELKAL, (**b**) ITAV_HUMAN – DPLEFKSH, (**c**) TNFL6_HUMAN–SLEKQIGH, (**d**) KNG1_HUMAN–SPFRSSRI, (**e**) FA8_HUMAN–RSKRALKQ, and (**f**) TNF13_HUMAN–RKRRAVLT.

In general, the addition of the inhibitor abolished the activity against a large number of peptide targets, indicating the predominance of SVMPs in the overall proteolysis. However, the venoms showed significant differences in the decrease of activity and the number of substrates that they cleaved (see Section 2.4). For example, activity from the original experiments was retained for a high number of peptides after the addition of metalloproteinase (MP) inhibitor to the venom of *E. carinatus*, while in the case of *B. asper* and *B. arietans*, the activity was considerably reduced. Examples of substrates against which the activity was abolished, or retained are shown in Figure 5, Figure 6 and Figure 7.

Many substrates that retained activity in the presence of the inhibitor were sequences derived from coagulation factors and inflammation mediators. Cleavage activity against peptides containing arginine residues was highly overrepresented.

Apart from the clear cases where the proteinase activity was either abolished or retained, certain substrates showed higher rates of turnover in the experiments with the inhibitor compared to the original experiments without the inhibitor, as can be seen in Figure 6d,f and Figure 7e. High cleavage activity against these substrates was observed in the different snake venom profiles tested with the MP inhibitor.

### 2.3. Models of Proteinase Activity

**Figure 8:**
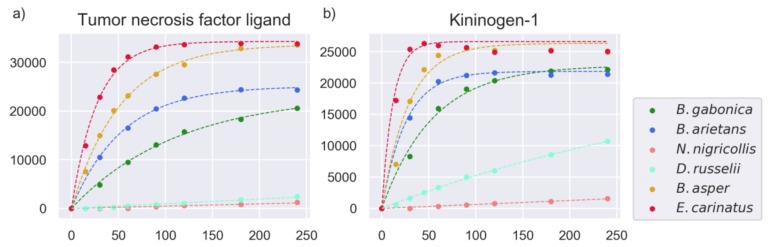
Selected substrates with fits of the model to experimental data. The model fits are represented as dashed lines, and the experimental fluorescence readings at different time points as scatter plots. The y-axis shows arbitrary fluorescence units (AFU), and the x-axis shows time (minutes). The common name of the protein of origin is shown as a title of each plot. The Uniprot IDs and amino acid sequences of these substrates were: (**a**) TNFL6_HUMAN–SLEKQIGH and (**b**) KNG1_BOVIN–SPFRSVQV.

The proteinase activity profiles were used to generate a model of the enzymatic activity of the proteinases in the snake venoms of the study. Nonlinear modeling was performed to estimate the parameters of cleavage activity by whole snake venom, using a model that has previously been utilized to describe proteinase kinetics [25,27,28] (for details, see Section 4.3). The 2160 rate constants calculated for all of the substrates across the six studied venoms will be referred to as *rates* in the remaining sections for simplification. Examples of the model’s fit with experimental data can be seen in Figure 8.

**Figure 9:**
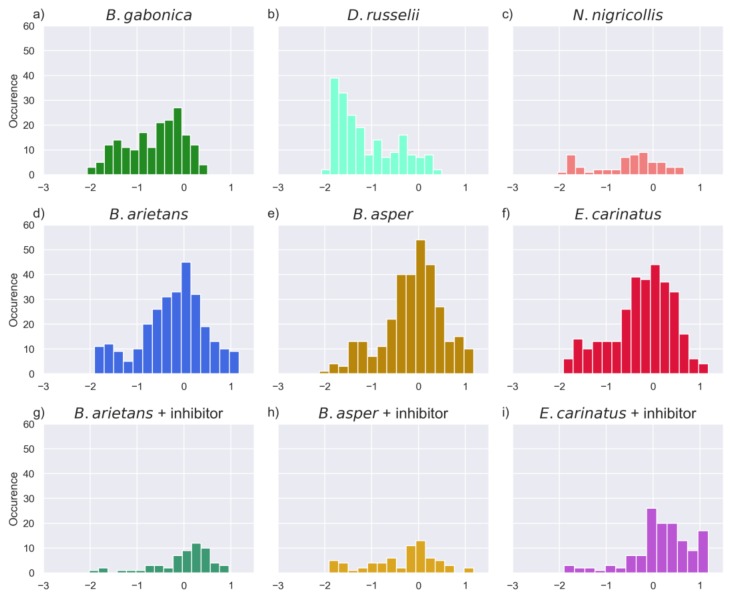
Histograms showing the distribution of the rates >1 × 10^−3^ across the six snake venoms subjected to investigation in this study, along with the inhibition experiments for the three snake venoms with the highest activity. Rates for substrates against which no activity was observed (rate <1 × 10^−3^) are not shown. The x-axis shows the rates in the log base 10 scale, while the y-axis shows occurrence (the number of substrates).

The parameter estimation was performed directly on the signal intensity measurements (AFU), with the rates being in units AFU/hour. Values above 0.1 indicate at least some substrate cleavage, and values above 1 indicate high and fast substrate turnover. Values below 0.01 indicate very low, or undetectable cleavage activity, as shown in Figure 1. The estimated rates for all of the substrates were used for further analysis to examine and compare the proteinase activity across the snake venoms of our study. Looking at the distribution of these rates, as shown in Figure 9, the venoms of *E. carinatus*, *B. arietans*, and *B. asper* exhibited the highest rates in the full substrate set, followed by *B. gabonica* venom, and then by *D. russelii* venom. The lowest rates were observed for *N. nigricollis* venom, as expected, as this elapid snake venom is known to have low proteinase content [29].

Experiments for *E. carinatus*, *B. asper*, *B. arietans*, and *D. russelii* venoms were replicated to assess the reproducibility of the cleavage activities measured in each substrate plate. In general, the replicated profiles were highly similar, with a low number of inconsistencies between them, e.g., false positives or false negatives. A comparison of the rate estimates showed a high Pearson correlation between all replicates (*E. carinatus* 0.93, *B. asper* 0.79, *B. arietans* 0.81, and *D. russelii* 0.71, also seen in Figure S1). For these four snake venoms, the rates estimated for each of the experiments for any given peptide were averaged (geometric mean) when used in further analyses (see Section 4.3 for details).

### 2.4. Substrate Cleavage Determination of Studied Venoms

In order to distinguish between cleaved and non-cleaved target peptides from the activities observed, cutoff values for both fluorescence signal intensity and the estimated rates were applied (see Section 4.4). Using these thresholds, a proposed number of cleaved substrates were determined for each venom tested. As shown in Table 1, *B. asper* venom cleaved the most substrates, followed closely by the venom of *E. carinatus*, and then by the venom of *B. arietans*, while the venoms of *B. gabonica*, *N. nigricolllis*, and *D. russelii* show much lower numbers of cleaved substrates. As shown in the previous subsection, although *N. nigricollis* has a few more cleaved substrates than *D. russelii* that passed the strict cutoffs imposed, the venom of *D. russelii* showed activity rates above the rate threshold for more substrates. Both of these venoms showed very low proteinase activity relative to the four other snake species.

**Figure 10:**
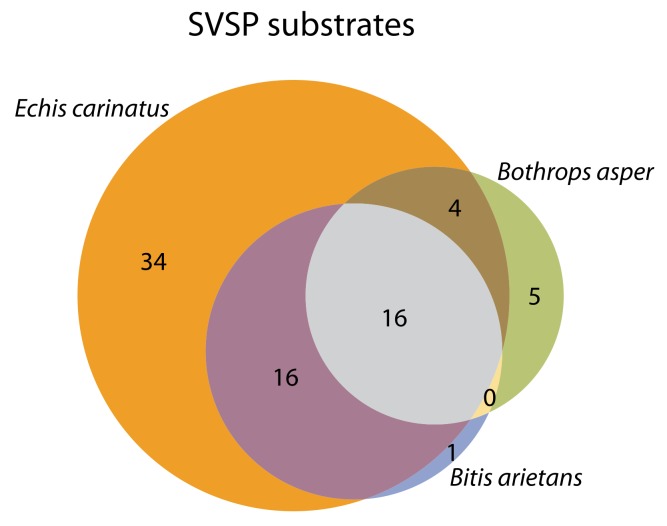
Venn diagram of the substrates that passed the cutoffs set, in the inhibitor-treated venom experiments. Since the activity of snake venom metalloproteinases (SVMPs) is inhibited in these experiments, these substrates are assumed to be targets of snake venom serine proteinases (SVSPs). *E. carinatus* shows the highest number of cleaved substrates, while many cleaved substrates are common between venoms. *B. arietans* and *B. asper* exhibit a low number of unique substrates.

It is noted that there was a small number of peptides (about half of them common between snake venoms) that did not pass the cutoffs in the original experiments, but did so in the inhibitor experiments. Consequently, the substrates cleaved in the inhibitor experiments are not entirely a subset of the substrates cleaved in the original experiments. A full list of peptide targets determined as cleaved for the snake venoms tested with and without the addition of the inhibitor, that could be potential metalloproteinase or serine proteinase substrates, can be found in File S1. The venom of *E. carinatus* retained 70% of its activity in the inhibition experiment compared to the original experiment, and was the venom with the highest activity in the inhibition experiments. On the other hand, the venoms of *B. arietans* and B. *asper* had approximately 40% and 20% of their original activity in the inhibition experiments. The substrates that passed the cutoffs imposed in the inhibition experiments, which were presumed to be SVSP substrates, were to a large extent common among the snake venoms, as can be seen in Figure 10.

### 2.5. Investigation of Relevant Targets for Human Snakebite Envenoming

**Figure 11:**
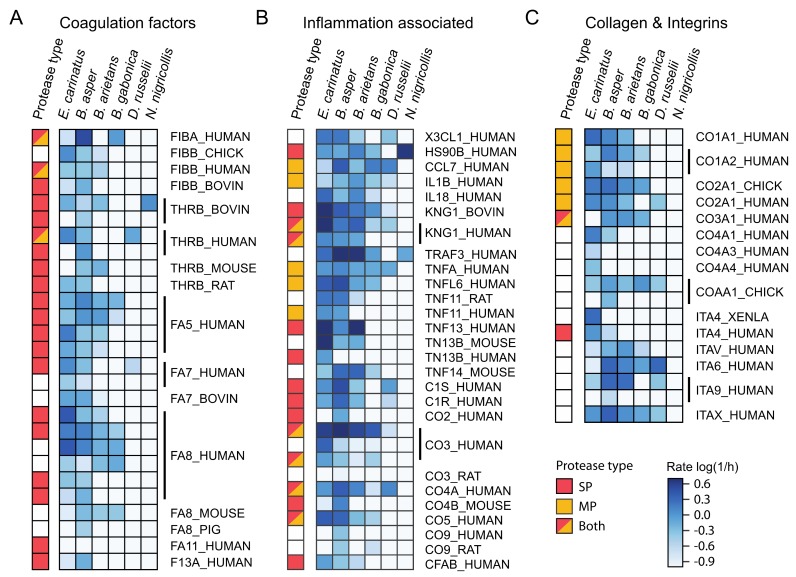
Heatmaps of coagulation factors (**A**), inflammation-associated proteins (**B**), and collagen and integrin-derived peptides (**C**) present in the substrate set used in the experiments. The cleavage activity for these substrates is indicated by the intensity of the respective box color. The scale of cleavage activity exhibited by the investigated venoms against these substrates is indicated by the estimated rate of substrate turnover, which is shown here as the base 10 logarithm of the rate. The substrates are identified by their Uniprot IDs. Substrates originating from the same protein are grouped together, as indicated by the vertical bars (substrate sequences not shown). The most left column (protease type) on the respective heatmap displays known cleavage sites in the substrate peptides for the two proteinase families extracted from the MEROPS database. It should be noted that MEROPS cleavage sites may be from any known metalloproteinase or serine proteinase, and not necessarily from snake venom proteinases.

As previously mentioned, the substrate set contained a number of target peptides derived from proteins that are or could be relevant for understanding envenoming pathophysiology. These substrates were grouped according to the biological process that they mediate, and the proteinase activity rates of the studied snake venoms were compared for these targets. Figure 11 shows heatmaps of substrates derived from relevant proteins in snakebite envenomings and their respective estimated rates for each snake venom tested. This method was employed to provide an overview of the snake venom proteinase activity in three distinct groups of substrates, namely coagulation-derived, inflammation mediator-derived, and collagen and integrin-derived substrate sequences. In addition, the presence of cleavage sites that are characteristic of metalloproteinases and serine proteinases in these peptide substrates are annotated in the figure.

The highest proteinase activity for proteins of the coagulation cascade was observed in the venoms of *E. carinatus*, *B. arietans*, and *B. asper*, although all of the viperid venoms showed activities against a wide range of peptides derived from coagulation factors. The highest activities were exhibited against substrates originating from coagulation factors V, VII, and VIII. A human prothrombin-derived substrate (IDGRIVEG) was highly cleaved when incubated with the venoms of *D. russelii* and *E. carinatus*, and by *B. asper* to a lesser extent. High activity was also observed against a human fibrinogen alpha chain peptide (GGVRGPRV) for the venoms of *B. gabonica* and *B. asper*. All of the venoms showed relatively low proteinase activity against a peptide (FSARGHRP) derived from human fibrinogen beta chain.

High proteinase activity was exhibited against substrates with inflammation mediator-derived sequences in a more uniform manner for all viperid venoms. The highest activities against all of these substrates were observed for the venoms of *E. carinatus*, *B. arietans*, and *B. asper*. *D. russelii* venom also had a considerable activity against peptides derived from inflammation-associated substrates, compared to the general low activity of this venom in the full substrate set. The most prominent target peptides with high cleavage rates were complement system components and tumor necrosis factor ligand substrates. High cleavage rates were also observed for substrates in other cytokines, such as human fractaline (AATRRQAV), C-C motif chemokine 7 (QPVGINTS), kininogen-1 (SPFRSSRI), and heat shock protein HSP 90-beta (DEEDDSGK).

High proteinase activity was also observed against peptides derived from collagen and integrin proteins (Figure 11). Significant signal intensity for all of the viperid venoms, except *D. russelii,* was observed against a peptide derived from human integrin alpha V (DPLEFKSH). High activity was also detected against peptides derived from human integrin alpha 6 (RPIPITAS), human integrin alpha X (RVLGLKAH), and integrin alpha 9 (VKRRVQDV). Viperid venoms showed high activity against collagen type I and collagen type II derived substrates. Collagen type IV-derived peptides were less labile to cleavage. Exceptions were observed, namely for the venom of *E. carinatus* on the peptide substrate derived from human collagen alpha 1 (IV) chain (GPPGIPGQ) and collagen alpha 3 (IV) chain (DGLPGLKG). Considerable activity was observed against the peptide substrate derived from *Gallus gallus* (chicken) collagen alpha 1 (X) chain (GPAGLSVL) and collagen alpha 1 (II) chain (RYMRADEA). In general, the venom of *E. carinatus* showed high activity against collagen and integrin substrates, followed by the venoms of *B. arietans* and *B. asper*.

The proteinase activity observed in the full substrate set in the inhibition experiments was greatly reduced compared to the original experiments, indicating that most of the proteinase activity in snake venoms is due to the action of SVMPs (Figure S2). However, *E. carinatus* venom retained high activity, especially against the substrates examined in this section. Most of the substrates that were cleaved in the inhibition experiments were peptides derived from coagulation factors and inflammation mediators. *E. carinatus* retained its activity in almost all of the peptides derived from coagulation factors V and VIII, while the peptides derived from coagulation factor VIII were susceptible to cleavage by all three venoms treated with MP inhibitor (*E. carinatus*, *B. asper*, *B. arietans*). Fibrinogen alpha chain (GGVRGPRV) was still cleaved by the venoms of *E. carinatus* and *B. asper*, while *B. arietans* showed no activity on this peptide with or without MP inhibitor. The high activity against a substrate derived from human prothrombin (IDGRIVEG) by the venom of *E. carinatus* was completely suppressed in the inhibition experiments. Proteinase activity was also lost against a peptide originating from bovine prothrombin (IEGRTSED), where all three venoms showed activity in the absence of an inhibitor.

Activity was retained against the fractaline substrate (AATRRQAV) for the venoms of *E. carinatus* and *B. arietans*, but not for the venom of *B. asper*. The activity against HSP90 beta (DEEDDSGK), IL1 beta (GPYELKAL), and CCL7 (QPVGINTS) substrates by all three venoms was abolished in the inhibitor experiments. On the other hand, activity against peptides derived from kininogen 1 human (SPFRSSRI) and bovine (SPFRSVQV) was still observed for all three venoms in the presence of an MP inhibitor. A human TNFA (LAQAVRSS) substrate was cleaved only by the venom of *E. carinatus* in the inhibition experiments, while a human TNF13 substrate (RKRRAVLT) was still highly cleaved by the venoms of *E. carinatus* and *B. arietans*. The activity against human complement C1r subcomponent (QRQRIIGG) was retained in all three venoms. The venom of *E. carinatus* kept its activity in all complement-derived substrates, while the venoms of *B. arietans* and *B. asper* lost their activity in all complement peptides, apart from the one mentioned. That was also the case in collagen and integrin substrates, where *B. arietans* and *B. asper* venoms lost their activity, and *E. carinatus* venom retained its activity in all but three examples (CO4A1, CO4A3, COAA1).

As previously mentioned, a significant number of substrates showed rates of substrate turnover that were higher in the inhibition experiments than in the original experiments. The substrates exhibiting higher rates were highly consistent across the three venoms tested with the addition of inhibitor. All of the substrates shown in Figure 11 against which activity was observed in the inhibition experiments contained arginine residues in their sequences, apart from one (IL18, LESDYFGK, for the venom of *E. carinatus*).

### 2.6. Comparison of Activities Reveals Phylogeny-Related Differences

In order to investigate the similarities and differences between the proteinase activities of the studied snake venoms, the Jaccard similarity coefficient was employed as a similarity measure. The estimated rates from the modeling of proteinase activity were discretized (see Section 4.5) to indicate whether cleavage activity was present or not.

**Figure 12:**
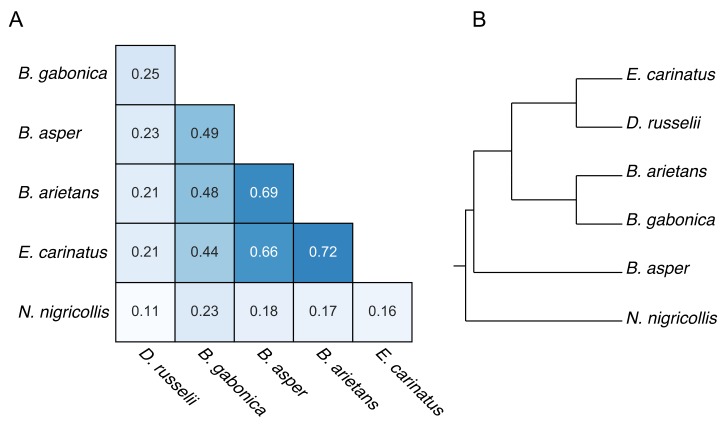
(**A**) Jaccard similarity comparing the activities of each snake venom against the rest of the studied venoms. (**B**) Phylogenetic tree showing relationship between the species of this study, adapted from [30].

As can be seen in Figure 12A, the highest similarities were observed for the venoms of *E. carinatus* with *B. arietans*, and *B. asper* with *B. arietans*. The tree (Figure 12B) shows the phylogenetic relationship of the studied snake species. The species of the *Bitis* genus are found to be closely related; the snake species of *D. russelii* and *E. carinatus* were grouped together, while the elapid *N. nigricollis* was an out-group, as anticipated. Relationships based on the Jaccard similarities observed between proteinase activities were considerably different than the phylogenetic relationship between these species (Figure 12).

## 3. Discussion

This study investigated the overall proteolytic activity of the venoms from the medically relevant snake species of two families from different regions of the world. The results of the high-throughput peptide screening analysis not only provide a general overview of the magnitude of proteinase activity for each snake venom, but also provide insights into relevant targets for the enzymes involved in the pathophysiology of snakebite envenomings. The high-throughput method was effective in investigating multiple candidate substrates of proteinases, instead of a single or limited number that can be tested by traditional spectrophotometric, SDS-PAGE, or zymography techniques [19,20,31,32,33]. Typically, the thorough characterization of the proteinase substrate requires a combination of electrophoretic techniques such as SDS-PAGE or 2D-DIGE, and mass spectrometry methodologies (LC-MS/MS, MALDI-TOF) [34,35,36,37,38], although recently developed MS techniques can be used without the need for an electrophoretic step [39,40,41]. However, these methods are quite laborious, compared to the substrate profiling method used in this study. Although this screening method does not provide the same amount of information in terms of cleavage site specificity, this technique is an adequate first step for the identification of proteinase substrates. In the context of snakebite envenoming, this study presents an easy, fast, and robust method to identify the targets of snake venom proteinases that could play a role in envenomings. Moreover, this approach allows for the identification of hitherto unknown substrates of snake venom enzymes, with possible implications for understanding the mechanism of action of venoms.

Cleavage activity was observed against substrates originating from known targets of snake venom proteinases, such as prothrombin, fibrinogen, other coagulation factors, integrins, and collagens. The interaction of these proteins with snake venom components is well described in the literature [42,43,44,45,46]. However, cleavage site locations and residues for these proteins have not previously been thoroughly investigated. Thus, method employed in this study could allow a more in-depth examination of substrates that are highly labile against snake proteinases in order to determine the specificity determining residues upstream and downstream of the cleavage sites. The same approach can be implemented for less described targets of snake venom proteinases. For example, the cleaved peptides with sequences derived from complement system components could be the primary step in the further investigation of the activity of proteinases on these proteins, and how these activities might affect envenoming pathology [47,48].

The proteolytic activity was analyzed using the experimental measurements and the modeling of said activity. When the full substrate set was considered, the highest activity was exhibited by *E. carinatus*, followed closely by *B. arietans* and *B. asper*. Interestingly, the activity of *B. arietans* venom showed more similarities with the above venoms compared to *B. gabonica* venom, which belong to the same genus. *D. russelli* venom showed lower overall proteolytic activity, but was more selective in terms of substrate cleavage, meaning that the activity observed reached higher values in these substrates. *N. nigricollis* venom exhibited the lowest proteinase activity, as expected from an elapid venom with different mechanisms of action and a low abundance of proteinases [29]. In general, the observed proteolytic activity indicated a strong relationship between the number of cleaved peptides and the overall proteinase abundance and the SVMP abundance in the snake venoms studied (Table 1 and Table 2).

In this relation, it is to be noted that wide intraspecies variations in snake venom compositions exist between specimens obtained from different geographical regions [58,59,60]. For the present analyses, proteinase abundances for the studied venoms were based on values reported in published venomics studies for representative species from geographical regions similar or identical to the region reported by the venom supplier [61]. More specifically, as can be seen in Table 2, *E. carinatus* venom contains a high abundance (about 60%) of snake venom metalloproteinases, with *B. asper* and *B. arietans* venoms having similar abundances (about 40%). The results of the inhibition experiments suggest that *E. carinatus* has the highest serine proteinase activity.

In general terms, proteinases in venoms play two main adaptive roles, i.e., toxicity leading to prey immobilization, killing, and digestion [9]. Proteinase-induced toxicity depends on the cleavage of selected targets of known physiological relevance, such as clotting factors, extracellular matrix proteins of the basement membrane of blood vessels, and precursors of inflammatory mediators, among others. *D. russelii* showed a reduced overall proteinase activity as compared to the other viperid venoms analyzed, in agreement with previous observations using azocasein as a substrate [50]. However, proteinases play a key role in the pathophysiology induced by this venom, as exemplified by an SVMP that activates factor X of the coagulation cascade, which is a key aspect in the hemostatic alterations characteristic of *D. russelii*-induced envenomings [62].

There is a number of described snake venom proteinases that could be responsible for the activities observed against certain relevant substrates. The MEROPS database provides an annotation of the substrates of proteinases. In some cases, the substrates of this study matched the sequence and protein of origin of substrates of these described proteinases, which makes them great candidates for explaining the activity observed in the experiments of this study. A P-III SVMP from *E. carinatus* venom, ecarin, is a known prothrombin activator. *E. carinatus* venom demonstrated high activity against a peptide (IDGRIVEG) derived from human prothrombin, which is among the known substrates of ecarin [63]. Another described metalloproteinase from *E. carinatus* venom, carinactivase, has a known target site in prothrombin, four residues upstream of the ecarin target site [64]. The activity against this human prothrombin substrate was completely abolished when the venom of *E. carinatus* was treated with *o*-phenanthroline, indicating that SVMPs were largely responsible for the substrate cleavage activity against this substrate. High proteinase activity was observed against the same prothrombin-derived substrate in our experiments for the venom of *D. russelii*. Another metalloproteinase from *E. carinatus* venom, lebetase, targets known substrate sequences in kininogen [65]. Partial sequences of these kininogen-derived substrates were among the substrates of this study (SPFRSSRI and SPFRSVQV), where *E. carinatus* venom showed the highest activity. For other peptides originating from prothrombin, the highest activities were observed for *E. carinatus* and *B. asper* venoms. *B. asper* venom also contains a known prothrombin-activating metalloproteinase, basparin, although no cleavage sites for this proteinase have been described [66]. *B. asper* venom contains another metalloproteinase with known fibrigenolytic activity called BaP1 [67]. The presence of this proteinase could explain the high activity that *B. asper* venom exhibited against a peptide derived from fibrinogen alpha chain (GGVRGPRV) in the experiments of this study. The same substrate was quite labile toward *B. gabonica* venom, which contains a serine proteinase called gabonase. This specific sequence of the fibrinogen alpha chain is a known substrate cleavage site of gabonase [68]. It is worth noting that two more known substrate sequences targeted by gabonase were among the substrates of this study, originating from fibrinogen beta chain (FSARGHRP) and factor XIII (VVPRGVNL). However, *B. gabonica* venom had little to no activity against these substrates. Lastly, a known factor X activating the metalloproteinase from the venom of *D. russelii*, russelysin, has annotated X factor sequences as its substrate cleavage sites [69]. Partial regions of this sequence (P1 and P’ residues) were found in substrates originating from factor VIII (PQGRIVGG), factor XI (IKPRIVGG), and kallikrein (TSTRIVGG). *D. russelii* venom showed considerable activity for these substrates.

Our observations on the degradation of peptides from various types of proteins playing distinct roles in physiology may provide clues to the action of these venom proteinases. Of particular interest is the hydrolysis of sequences of proteins involved in the coagulation cascade or inflammation, those that are part of the extracellular matrix, and integrins. Regarding proteins of the clotting cascade, heatmap analysis showed that the venoms of *E. carinatus*, *B. asper, B. arietans*, and *B. gabonica* showed the highest activity, followed by *D. russelii*. From a functional standpoint, the proteolysis of a clotting factor may result in two different outcomes: (a) the factor may be activated, as in the case of SVMPs that activate factor X or prothrombin, and SVSPs that convert fibrinogen into fibrin [70], or (b) the factor might be degraded to an inactive form. For an example of the latter, two of the peptides in this study that were derived from coagulation factors V and VIII are known to contain the cleavage sites of activated protein C, which is an anticoagulant proteinase among others (these two substrates were highly cleaved by *E. carinatus* and *B. arietans*, and to a lesser extent by *B. asper*). These two actions have highly different pathophysiological consequences. This duality is evident when analyzing the heat maps of coagulation cascade proteins *vis-à-vis* the in vitro coagulant activity of venoms (Table 2). The venoms of the two *Bitis* species are able to degrade several peptides from clotting factors without being coagulant in vitro, whereas the venom of *D. russelii* cleaves a fewer number of substrates, and yet has in vitro clotting activity. Likewise, the hydrolysis of proteins playing roles in the inflammatory response may be associated with the cleavage of a precursor and the release of an active component, such as the case in the hydrolysis of pro-tumor necrosis alpha (TNF) described for the venom of *Echis ocellatus* [71] or the activation of complement factors [72]. In contrast, the hydrolysis of inflammatory mediators may result in inactivation. Hence, the widespread hydrolysis observed for sequences of a number of inflammatory mediators by venom proteinases may play positive and negative modulatory roles in the inflammatory response.

Analysis of the cleavage sites of extracellular matrix proteins and integrins also revealed higher collagenolytic activity in the venoms displaying an overall higher proteinase activity, i.e., *B. asper*, *E. carinatus*, and the two species of *Bitis*. Collagen hydrolysis has been associated with the degradation of the extracellular matrix, which characterizes the severe local tissue pathology that is characteristic of viperid snakebite envenoming induced by the action of SVMPs [73]. There is a relationship between the number of collagen-derived peptides cleaved and the hemorrhagic activity of venoms (Table 2). Among viperid venoms, *D. russelii* shows the lowest number of cleaved peptides and the lowest hemorrhagic activity, whereas the venom of *N. nigricollis* is not hemorrhagic and cleaves few collagen-derived peptides. The hydrolysis of peptides with sequences derived from integrins may also have implications for the actions of these venoms, as these proteins play key roles in cell–cell and cell–extracellular matrix interactions. In particular, the high activity observed in four viperid venoms against integrin alpha V, and in the light of the viperid venoms inducing skin blistering, may have implications due to the known role of this integrin in the skin [74]. The observation of the hydrolysis of peptides with sequences derived from intracellular proteins may bear functional implications on two grounds. (a) Upon cell death by the action of venom cytotoxins, intracellular components are exposed, and therefore prone to hydrolysis by venom proteinases, thus contributing to the overall digestion of tissues. (b) Some products of proteolysis may acquire functional roles of various sorts, becoming mediators of cellular activation or constituting damage-associated molecular patterns (DAMPs), which participate in the overall tissue response to venoms [75]. Among the substrates tested in this work, HSP 90 beta (DEEDDSGK) has been described as a DAMP [75].

The proteinase activity against the substrates studied exhibited different steady-state levels (level of fluorescence signal where the activity seems to be saturated), as measured by the fluorescence intensity measurements. The different steady-state levels might be explained by the aggregate effect of a different number of proteinases that are responsible for the cleavage possess of the specific substrate sequences in the same venom, differences in the amount of labeled peptide loaded in each well, or inadequate duration of the assay for the given target peptide to reach its steady-state level. In some cases, a drop-off in fluorescence readings was observed at later time points. This drop-off from the maximum fluorescence measured in earlier stages could be caused by re-quenching of the fluorophores by free quenchers in the solution, or by photobleaching, rendering the fluorophores unable to fluoresce. The observation of higher rates for a number of substrates in the experiments where the metalloproteinase inhibitor phenanthroline was added is intriguing, and cannot easily be explained. However, speculations on the cause of this phenomenon can be mentioned. It is possible that the SVMPs were limiting the availability of a given substrate for cleavage by serine proteinases, by binding to the substrate, or preventing it from binding to serine proteinases in the original experiments. Inhibiting SVMPs with the chelator phenanthroline, and subsequently destabilizing them by the loss of the metal ion, would eliminate the scenario, resulting in higher rates for the substrates of serine proteinases. Another possible explanation could be that the serine proteinase activity was limited in the original experiments, but not in the inhibition experiments. Moreover, the occurrence of higher rates for certain substrates in the inhibition experiments would be attained if SVMPs from the snake venoms were cleaving, and thereby inactivating serine proteinases in the original experiments. Finally, it might be possible that other snake venom components were affecting the cleavage activity of serine proteinases on substrates, and that the inhibition of the serine proteinase activity was removed in the inhibition experiments by the interaction of these assumed components with the inhibitor.

The cleavage detection method of this study proved to be an adequate indicator of proteinase cleavage activity and a useful tool for comparing the snake venom proteinase activities against a standardized set of targets. Expanding this datasets by the screening of more snake venoms would provide a wider framework for the investigation of the scale of proteinase activity in these venoms, as well as allow the examination of the differences and similarities in these activities. This method of cleavage detection also shows promise in the identification and discovery of targets of specific proteinases. By incubating purified proteinases with selected peptide targets of interest, several potentially relevant targets of these proteinases could be screened, and the proteinase activity against these substrates could be assessed. Moreover, this method could present significant value outside of the boundaries of snakebite envenoming research. The determination of substrate cleavage for proteins of clinical importance in other fields could demonstrate the possibility of utilizing specific snake venom proteinases as ‘tools’ for scientific research and drug development in these disciplines [76,77].

## 4. Materials and Methods

### 4.1. Snake Venoms

Snake venoms of interest were purchased in lyophilized form (Latoxan S.A.S., Valence, France). The venoms of *B. gabonica* (L1104A, Burundi), *B. arietans* (L1159A, Tanzania), *N. nigricollis* (L1327B, Tanzania), *D. russelii* (L1132A, Pakistan), *E. carinatus sochureki* (L1111, Pakistan), and *B. asper* (L1209B, Costa Rica) were obtained as pooled samples from several specimens.

### 4.2. Proteinase Activity and Inhibition Experiments

The enzymatic activity of each venom was tested using the Protease Substrate Set (JPT Peptide Technologies GmbH, Berlin, Germany). The JPT Peptide Technologies Protease Substrate Set is a 384-well plate with 360 different 8-mer peptides derived from natural protein cleavage sites described in the scientific literature. The remaining 24 wells are reserved for controls. The 360 peptides are flanked by a quencher molecule (DABCYL) and a fluorophore Glu(EDANS)-amide at the N-terminus and the C-terminus, respectively. These two moieties are separated upon peptide cleavage, allowing the EDANS molecule to fluoresce. Brij L23, 30% *w*/*v* (B4184, Sigma-Aldrich, St. Louis, MO, USA) was diluted to 0.03% *w*/*v* and used for the suspension of the freeze-dried peptides in the substrate set (10 μL per well). The venoms were resuspended to a starting concentration of 300 μg/mL in 0.03% *w*/*v* Brij solution. Then, 5 μL of venom suspension was added to each plate well for a final concentration of 100 μg/mL. The concentration for the peptide substrates in the wells was 5 μM at a final volume of 15 μL. In each experiment, nine wells were loaded with control solutions: 3 wells with Brij solution (0.03% *w*/*v*), 3 wells with venom (300 μg/mL), and 3 wells with venom (100 μg/mL) and Brij solution (0.03% *w*/*v*) at a volume of 15 μL. Fluorescence was measured using a Cytation 5 Imaging Reader (BioTek Instruments, Winooski, VT, USA) at different time points, with excitation at 350 nm and emission at 490 nm, according to the plate manufacturer’s instructions. For the venoms of *B. gabonica*, *B. arietans,* and *N. nigricollis* measurements were collected at seven different time points: 0 (background), 30, 60, 90, 120, 180, and 240 min. For the venoms of *D. russelii*, *B. asper,* and *E. carinatus*, additional measurements were taken at 15 and 45 min for greater resolution in the early stages of the enzymatic activity.

The metalloproteinase inhibition experiments were conducted following the same protocol as described above, with the exception that the metalloproteinase inhibitor was added in the venoms after their suspension. The metalloproteinase inhibitor 1,10-phenanthroline (P9375, Sigma-Aldrich) was dissolved in methanol and diluted to a 200 mM stock solution. The inhibitor was added to the suspended venoms and the solution was incubated at 37 °C for 30 min, with the final inhibitor concentration of 10 mM in the substrate plate wells. The pH of the venom solution was not affected by the addition of the inhibitor. The control solution triplicates used were the same as above, with the addition of phenanthroline in the suspended venoms. Measurements were taken for the background signal intensity and eight different time points, as mentioned above. The raw data obtained from all of the experiments are available in File S2.

### 4.3. Data Analysis and Modeling

The analysis of the experimental data was conducted with Python 3.6 [78], using the Python packages Numpy 1.13.3 [79], Pandas 0.20.3 [80]. Matplotlib 2.1.0 [81] and Seaborn 0.9.0 [82] were used for data visualization and figure generation. Kinetic parameter estimation was performed using the function optimize.curve_fit (Scipy 0.19.1 [83]), and statistical significance tests were carried out with the Scipy and StatsModels 0.8.0 [84] packages.

Relative fluorescence graphs were constructed by the subtraction of the base-2 logarithm values of the background measurements from the base-2 logarithm values of the later time points, for each well signal intensity. The following equation was used to describe product accumulation:

*P*(*t*) = *P*_max_(1 − *e*^−*αt*^) (1)

which has previously been used to describe proteinase kinetics in experiments utilizing fluorescence assays [25], and phage substrate methods [27], along with equations to convert fluorescence in FRET assays to substrate concentration [28]. This equation was adjusted to allow for different levels of background fluorescence (*F*_min_) and different maximum fluorescence levels (*F*_max_). Thus, the equation used to model proteinase activity was the following:

*F*(*t*) = (*F*_max_ − *F*_min_) × (1 − *e*^−*λt*^) + *F*_min_ (2)

where the *F*_max_, *F*_min_, and the rate constant λ were estimated for all of the proteinase activity profiles. The *F*_min_ and *F*_max_ parameters were initialized using the minimum and maximum fluorescence values observed. An initial value of 10^−5^ was used for the rates, indicating an activity level well below detection. Initialization using different λ values did not affect the final estimations. In cases where the background measurement (*F*_min_) was slightly higher than in later time points (mostly evident in *N. nigricollis* venom substrate activity profile), overestimation of the rate constants was observed. For that reason, the background estimates were subtracted from all of the fluorescence measurements, and negative values at later measurements (in the rare cases that the signal intensity was slightly lower than the background measurements) were set to zero. Consequently, the *F*_min_ of the model was also zeroed. In the inhibition experiments, the fluorescence signal emission caused by phenanthroline was subtracted from the measurements, using the average of the triplicate controls that were present. The estimation of the parameters and model fitting was done after the measurements were adjusted for the fluorescence drop-off of the later measurements, in the limited instances that this phenomenon occurred. Lower and upper boundaries of 0 and 15 AFU/hour were used for λ estimation. Wells that showed maximum fluorescence intensity below the arbitrary cutoff of 5000 AFU were disregarded as having negligible or no activity, and the rates of those wells were reinitialized and not used for subsequent analysis. In the cases where duplicates of the experiments existed, the rates estimated for each of the experiments for any given peptide were averaged using the geometric mean of the two values. The estimated rates for all of the peptide targets across all of the venoms can be found in File S3.

### 4.4. Substrate Cleavage

For a given substrate to be deemed as cleaved by a certain venom, two cutoffs had to be satisfied: one for the estimated rates and one the fluorescence signal intensity, respectively. A cleavage rate above 1 (10^0^) and a fluorescence measurement value of above 50% of the 95^th^ percentile value of the fluorescence signal observed on each substrate plate (to account for the variances of fluorescence signal in the different plates) was needed. These cutoffs had to be satisfied in the first two hours of measurements.

### 4.5. Jaccard Similarity Coefficient and Phylogeny

After analyzing the estimated model parameters, it was apparent that for each comparison, the rates could be grouped in four different categories. For each substrate, these were: cleavage activity by both snake venoms, negligible or no activity for either, activity for one and not for the other, or vice versa. The Jaccard coefficient was employed to assess the similarities between venom activities, as it has been used successfully to describe various biological interactions and processes that could be classified as binary [85,86]. The model rate parameters were discretized into two ranges of values (10^−5^ to 10^−1^ and 10^−1^ to 15), making it possible to classify them into undetectable or very low activity (assigned zero) and considerable activity (assigned one) cleavage using a cutoff value of 10^−1^. The Jaccard coefficient was calculated using the equation:

J = R11/(R11 + R10 + R01)(3)

where R11 is the number of the discretized rates assigned one for both snake venoms, and R10 and R01 are the number of discretized rates that were assigned one for one of the snake venom activities and zero for the other, respectively.

The phylogenetic tree of the snake species in this study was created by pruning of the original phylogenetic tree constructed in [30], retaining only the species of interest from the newick file of the referenced study. Pruning was conducted in Python 2.7 using the objects and the prune function of the ete2 [87] phylogenetic analysis package. The distances between species were omitted for better visualization.

## Figures and Tables

**Table 1:** Quantification of substrate cleavage across the studied snake venoms, in absolute number of substrates and as a percentage of the full substrate set. The cleaved substrates in the experiments where the venoms are treated with the inhibitor phenanthroline (PT) are shown on the right.

Venom	Substrates Venom	% of Substrates	Substrates Venom + PT	% of Substrates
*B. asper*	98	27.2	21	5.8
*E. carinatus*	95	26.3	70	19.4
*B. arietans*	76	21.1	32	8.8
*B. gabonica*	15	4.1		
*D. russelii*	8	2.2		
*N. nigricollis*	13	3.6		

**Table 2:** Relative abundance of snake venom proteinases in the studied venoms, according to proteomic studies, along with a quantification of hemorrhagic and coagulant activities of the venoms (minimum venom dose required to induce hemorrhage and coagulation, respectively), as described in the indicated references. SVSPs: snake venom serine proteinases.

Venom	Proteinase%	SVMPs%	SVSPs%	Min. Hemorrhagic Dose (μg)	Min. Coagulant Dose (μg)
*E. carinatus*	61.2 [49]	56.6	4.6	0.30 [50]	3.30 [50]
*B. asper*	59.2 [51]	41.0	18.2	1.50 [52]	0.32 [52]
*B. arietans*	57.9 [53]	38.5	19.5	0.15 [54]	Non-coagulant
*B. gabonica*	54.7 [55]	30.8	23.9	0.38 [54]	Non-coagulant
*D. russelii*	25.0 [56]	21.8	3.2	4.30 [50]	4.00 [50]
*N. nigricollis*	2.4 [29]	2.4	-	Non-hemorrhagic [57]	Non-coagulant

## Supplementary Materials

The following are available online at https://www.mdpi.com/2072-6651/11/3/170/s1, Figure S1: Correlation plots of replicate experiments for the snake venoms of *B. arietans*, *B. asper*, *E. carinatus*, and *D. russelii*, Figure S2: Heatmaps of coagulation factors (A), inflammation-associated proteins (B), and collagen and integrins (C) comparing the proteinase activity in the selected peptide targets in the original experiments and the experiments with the addition of inhibitor, File S1: Excel file containing assumed metalloproteinase substrates and assumed serine proteinase substrates for the snake venoms of *B. arietans*, *B. asper*, and *E. carinatus*, File S2: Excel file containing all of the fluorescence intensity measurement data from all of the experiments conducted, File S3: Excel file containing all of the estimated rate constants for all of the snake venom experiments conducted in this study.
