# Supplementary material for: Protease Activity Profiling of Snake Venoms Using High-Throughput Peptide Screening
Source: Toxins (Basel). 2019 Mar 19;11(3):170. doi: 10.3390/toxins11030170 (PMC6468401; doi:10.3390/toxins11030170)
Supplement: Supplementary file 1 [file toxins-11-00170-s001.zip › toxins-463104-final-supplementary/toxins-463104-final-supplementary.docx]

Supplementary Materials: Protease Activity Profiling of Snake Venoms Using High-Throughput Peptide Screening

Konstantinos Kalogeropoulos, Andreas Frederik Treschow, Ulrich auf dem Keller, Teresa Escalante, Alexandra Rucavado, José María Gutiérrez, Andreas Hougaard Laustsen
and Christopher T. Workman *


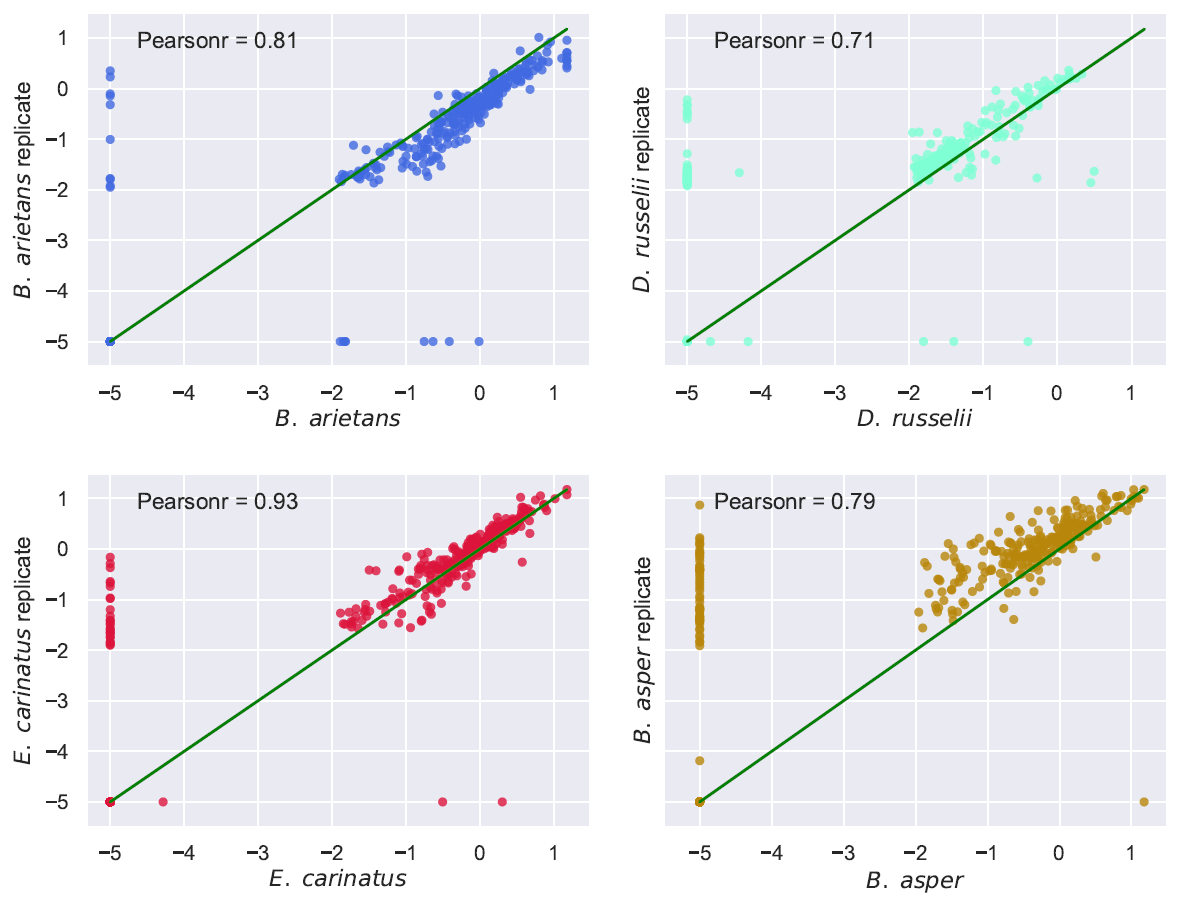


**Figure S1.** Correlation plots of replicate experiments for the snake venoms of *B. arietans*, *B. asper*, *E. carinatus*, and *D. russelii*.


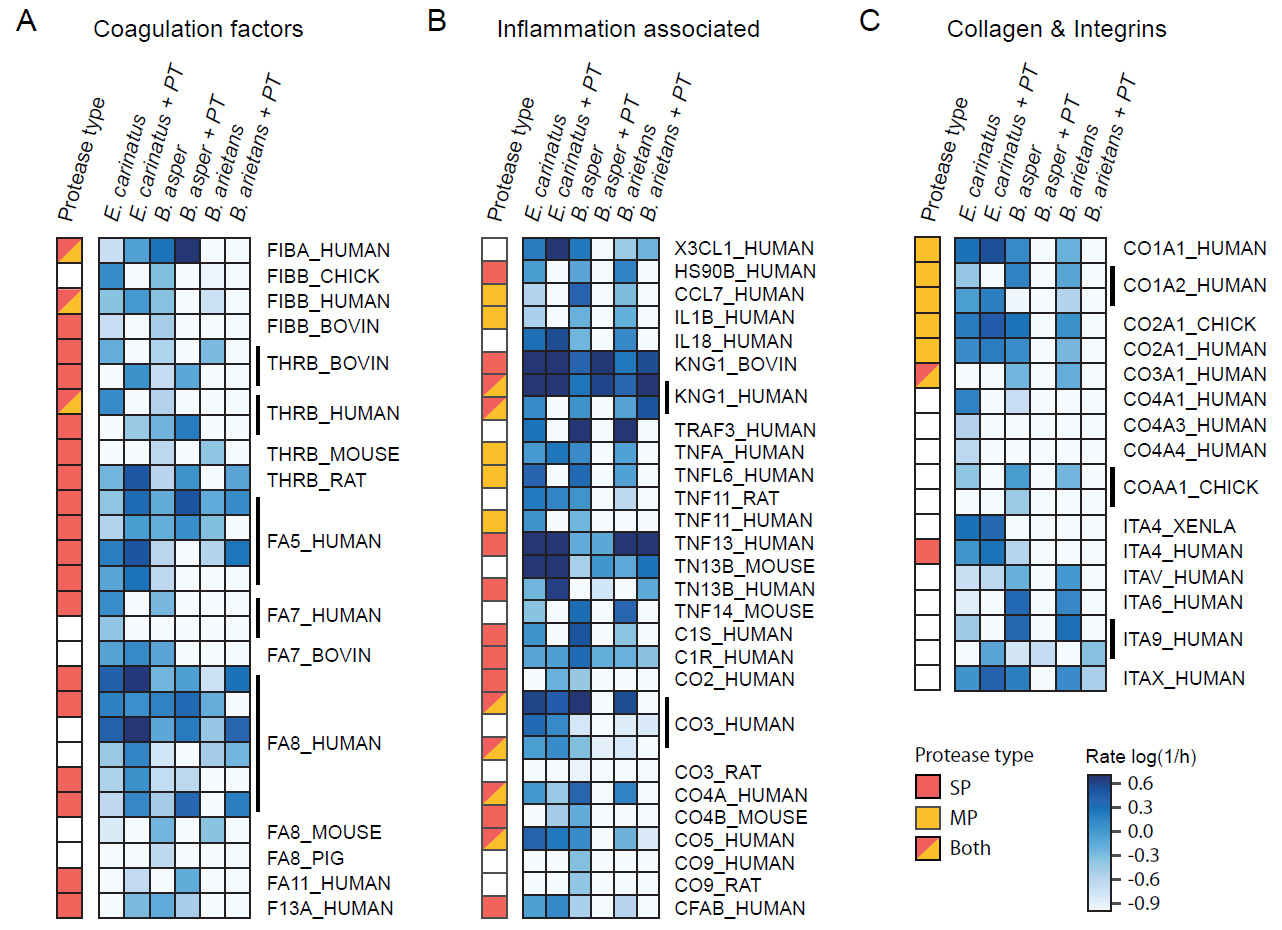


**Figure S2.** Heatmaps of coagulation factors (**A**), inflammation associated proteins (**B**), and collagen and integrins (**C**) comparing the proteinase activity in the selected peptide targets in the original experiments and the experiments with the addition of inhibitor.
